# Prostate cancer cells synergistically defend against CD8 ^+^ T cells by secreting exosomal PD‐L1

**DOI:** 10.1002/cam4.6275

**Published:** 2023-07-27

**Authors:** Dameng Li, Xueying Zhou, Wenxian Xu, Yuxin Chen, Chenglong Mu, Xinchun Zhao, Tao Yang, Gang Wang, Liang Wei, Bo Ma

**Affiliations:** ^1^ Cancer Institute Xuzhou Medical University Xuzhou China; ^2^ Center of Clinical Oncology The Affiliated Hospital of Xuzhou Medical University Xuzhou China; ^3^ Jiangsu Center for the Collaboration and Innovation of Cancer Biotherapy, Cancer Institute Xuzhou Medical University Xuzhou China

**Keywords:** anti‐PD‐L1 therapy, CD8^+^ T cells, exosomes, PD‐L1, prostate cancer

## Abstract

**Background:**

Metastatic castration‐resistant prostate cancer (mCRPC) remains fatal and incurable, despite a variety of treatments that can delay disease progression and prolong life. Immune checkpoint therapy is a promising treatment. However, emerging evidence suggests that exosomal programmed necrosis ligand 1 (PD‐L1) directly binds to PD‐1 on the surface of T cells in the drain lineage lymph nodes or neutralizes administered PD‐L1 antibodies, resulting in poor response to anti‐PD‐L1 therapy in mCRPC.

**Materials and Methods:**

Western blotting and immunofluorescence were performed to compare PD‐L1 levels in exosomes derived from different prostate cancer cells. PC3 cells were subcutaneously injected into nude mice, and then ELISA assay was used to detect human specific PD‐L1 in exosomes purified from mouse serum. The function of CD8^+^ T cells was detected by T cell mediated tumor cell killing assay and FACS analysis. A subcutaneous xenograft model was established using mouse prostate cancer cell RM1, exosomes with or without PD‐L1 were injected every 3 days, and then tumor size and weight were analyzed to evaluate the effect of exosomal PD‐L1.

**Results:**

Herein, we found that exosomal‐PD‐L1 was taken up by tumor cells expressing low levels of PD‐L1, thereby protecting them from T‐cell killing. Higher levels of PD‐L1 were detected in exosomes derived from the highly malignant prostate cancer PC3 and DU145 cell lines. Moreover, exosomal PD‐L1 was taken up by the PD‐L1‐low‐expressing LNCaP cell line and inhibited the killing function of CD8‐T cells on tumor cells. The growth rate of RM1‐derived subcutaneous tumors was decreased after knockdown of PD‐L1 in tumor cells, whereas the growth rate recovered following exosomal PD‐L1 tail vein injection. Furthermore, in the serum of mice with PCa subcutaneous tumors, PD‐L1 was mainly present on exosomes.

**Conclusion:**

In summary, tumor cells share PD‐L1 synergistically against T cells through exosomes. Inhibition of exosome secretion or prevention of PD‐L1 sorting into exosomes may improve the therapeutic response of prostate tumors to anti‐PD‐L1 therapy.

## 1 INTRODUCTION

Prostate cancer is the second highest cause of cancer deaths among men globally.^1^, ^2^ Most deaths are from metastatic castration‐resistant prostate cancer (mCRPC), which is independent of androgen for growth and survival, leading to resistance to multiple treatments.^3^, ^4^, ^5^, ^6^, ^7^, ^8^ Although immunotherapies are at the forefront due to unprecedented durable response rates in specific cancer types, literature has so far failed to provide convincing benefit of immunotherapy.^9^, ^10^, ^11^ Only a small subset of selected subgroup of mCRPC patients can benefit from anti‐PD‐L1 therapy.^9^, ^12^, ^13^ Thus, it is important to decipher the underlying mechanisms of mCRPC resistance to anti‐PD‐L1 immunotherapy.

Programmed death ligand 1 (PD‐L1) is a transmembrane protein that binds to programmed death receptor 1 (PD‐1) on immune T cells. Upon their binding, SHP2 is activated to dephosphorylate TCR and CD28, which subsequently inhibits the proliferation and activation of CD8^+^ T cells. The PD1/PD‐L1 axis is responsible for cancer immune escape and has a great impact on cancer therapy.^14^, ^15^ It is generally accepted that PD‐L1 relies on direct contact between tumor cells and immune cells to exert its function. However, emerging evidence suggests that prostate cancer cells can suppress the function of T cells in draining lymph nodes or block anti‐PD‐L1 antibodies by secreting exosomal PD‐L1 into the system.^16^, ^17^ Thus, exosomal PD‐L1 is critical for resistance to anti‐PD‐L1 immunotherapy in prostate cancer.

Exosomes are biologically active lipid‐bilayer nanovesicles with a diameter of 30–150 nm that enter the extracellular space through the endosomal pathway.^18^ As an important mode of intercellular communication, exosomes can deliver content from donor cells to recipient cells including proteins, RNA, DNA, and lipids.^19^, ^20^, ^21^ In the tumor microenvironment, tumor cells frequently utilize exosomes for intercellular communication to share microRNAs, lncRNAs, and proteins such as EGFR.^19^, ^22^, ^23^, ^24^ However, it is unclear whether prostate cancer cells share PD‐L1 through exosomes to synergistically achieve immune escape.

Herein, we found that prostate cancer cells with high PD‐L1 expression secreted exosomal PD‐L1 into the system, which was then taken up by prostate cancer cells with low PD‐L1 expression, protecting them against T‐cell killing and promoting tumor progression.

## 2 MATERIALS AND METHODS

### 2.1 Mice

C57BL/6J and nude male mice were purchased from GemPharmatech. Mice were housed in SPF facilities. All animal procedures were conducted in accordance with the guidelines of the Institutional Animal Care and Use Committee of Xuzhou medical university.

To construct the human prostate cancer xenograft model in nude mice, PC3 cells (1 × 10^6^ cells in 100 μL medium) were injected subcutaneously into the flanks of 8‐week‐old male nude mice. Mice were euthanized 3 weeks after tumor implantation or when the longest dimension of the tumor reached 2 cm. After anesthesia, blood samples were obtained by cardiac puncture.

To construct the mouse prostate cancer model in C57BL/6J mice, RM1 cells or RM1 PD‐L1^KO^ cells (1 × 10^6^ cells in 100 μL medium) were injected subcutaneously into the flanks of 8‐week‐old male C57BL/6J mice. Exosomes (PD‐L1^HA^ or PD‐L1^KO^, 100 μg in 100 μL PBS) were injected into the tail vein every 3 days. Mice were euthanized 3 weeks after tumor implantation. Tumor size was measured using vernier calipers, and the tumor volume was calculated using the following formula: (width)^2^ × length/2.

### 2.2 Cell culture

PC3, LNCaP, 22RV1 human prostate cancer cell lines were cultured in RPMI 1640 medium supplemented with 10% fetal bovine serum (FBS). DU145 cells were cultured in DMEM with 10% FBS. The RM1 mouse prostate cancer cell line was cultured in RPMI 1640 medium with 10% FBS.

### 2.3 Plasmids

The plasmids encoding pcDNA3.1‐HA‐PD‐L1 (human), pcDNA3.1‐eGFP‐PD‐L1 (human), and pcDNA3.1‐HA‐PD‐L1 (mouse) were purchased from GenScript Co. Ltd. The PD‐L1 protein was labeled with HA or eGFP.

For depletion of PD‐L1 in PC3 or RM1 cells, the sgRNA oligonucleotides were cloned into pLentiCRISPR V2. For gene depletion, two different guides were transfected into PC3 or RM1 cells using Lipo2000 (Table 1). After 48 h, the cells were selected with 1 μg/mL puromycin. After sorting, single live cells were cultured in 96‐well plates. PD‐L1 knockout efficiency was detected using western blotting.

**TABLE 1:** sgRNA for PD‐L1 depletion.

sgRNA oligonucleotides	Sequences
human *Pd‐l1* guide 1	ACCGTTCAGCAAATGCCAGT
human *Pd‐l1* guide 2	TCTTTATATTCATGACCTAC
mouse *Pd‐l1* guide 1	GTTTACTATCACGGCTCCAA
mouse *Pd‐l1* guide 2	GGGGAGAGCCTCGCTGCCAA

### 2.4 ELISA

The human PD‐L1 ELISA kit (Proteintech; KE00074) was used to detect and quantify protein levels of PD‐L1. In brief, 100 μL serum or exosomes purified from 100 μL serum were added to each well, which had been pre‐coated with an antibody specific for PD‐L1. After incubation, extensive washing was performed. Then, another antibody specific for PD‐L1 was used to detect the captured PD‐L1 protein. Following incubation and extensive washing, an HRP‐conjugated antibody was added for signal development. For color development, TMB reagent was added, followed by a stop solution containing sulfuric acid. The color intensity was measured at 450 nm with the correction wavelength at 630 nm.

### 2.5 Exosome isolation

PC3, DU145, LNCaP, 22RV1, and RM1 cells were cultured in 15 cm plates for 48 h. The culture medium was collected, and centrifuged at 300 ***g*** for 5 min, 2000 ***g*** for 20 min, and then 10,000 ***g*** for 30 min. The supernatant was filtered through 0.22 μm syringe filters. The exosomes were concentrated by ultracentrifugation at 110,000 ***g*** for 70 min. The exosome pellets were re‐suspended in PBS.

The mouse serum was centrifuged at 2000 ***g*** for 30 min, then 0.2 volumes of VEX Exosome Isolation Reagent (from serum, vazyme) were added. Exosomes were collected according to the manufacturer's protocol and then re‐suspended in PBS. TEM photography services were provided by Servicebio Technology Co. Ltd. Particle size distribution was measured using a Zetasizer Nano ZS 90 (Malvern).

### 2.6 T‐cell‐mediated tumor cell killing assay

To acquire CD8^+^ T cells, PBMCs were activated with IL‐2 (10 ng/mL) and CD3/CD28 (100 ng/mL). After co‐culture of tumor cells and T cells in 24‐well plates with or without exosomes containing PD‐L1 for 5 days, the wells were washed three times with PBS to remove T cells. Tumor cells in 24‐well plates were fixed with 4% paraformaldehyde, stained with crystal violet solution, and photographed.

### 2.7 Detection of cytokine secretion function of CD8 ^+^ T cell

The CD8^+^ T cells were stimulated with PMA (50 ng/mL) and ionomycin (1 μM) for 6 h and added Brefeldin A (2 μg/mL) at the same time. The percentage of CD3^+^, CD8^+^, Granzyme A+, Granzyme B+, TNF‐α+, IFN‐γ + cells were detected by flow cytometry. These proteins were stained by fluorescently labeled primary antibody as follows: Percp/cy5.5‐CD3 (Invitrogen, 45–0037‐42), PE/cy5‐CD8 (Invitrogen, 15–0088‐42), AF647‐Granzyme A (Biolegend, 507214), PE‐Granzyme B (Invitrogen, GRB04), PE/cy7‐TNF‐α (Invitrogen, 25–7319‐82), FITC‐IFN‐γ (Invitrogen, 11–7319‐82). The cytokines were stained with antibodies after fixation and permeabilization using BD Cytofix/Cytoperm (BD, 554714). Experiments were performed on BD LSRFortessa. Data were analyzed by FlowJo.

### 2.8 Western blotting

Samples were lysed in RIPA buffer (Beyotime; P0013B) with the addition of protease and phosphatase inhibitors. For protein concentration detection, the BCA protein assay kit (Thermo Fisher Scientific; 23225) was used. Primary antibodies included CD9 (Proteintech; 20,597); Alix (Proteintech; 12422), TSG101 (Proteintech; 28283), and PD‐L1 (Proteintech; 66248). For the development of the blots on NC membranes, ECL reagents (Thermo Scientific, 34580) were used.

### 2.9 Immunofluorescence

For Immunofluorescence staining of PC3 or DU145 cells, cells transfected with pcDNA3.1‐eGFP‐PD‐L1 were seeded and cultured overnight, then fixed with 4% paraformaldehyde for 30 min. After blocking with 2% BSA, cells were incubated with CD63 antibody (1:100; Santa Cruz; sc5275) overnight at 4°C, followed by goat anti‐mouse Alexa Fluor 594 (1:500; Invitrogen; A11005) secondary antibody incubation at room temperature for 1 h. The nuclei were stained with DAPI. Samples were imaged using an Olympus microscope at 60× magnification.

LNCaP cells were co‐cultured with exosomes‐PD‐L1^eGFP^ derived from DU145 or PC3 cells and fixed with 4% paraformaldehyde for 30 min. The nuclei were stained with DAPI. Samples were imaged using an Olympus microscope at 60× magnification.

### 2.10 Statistical analyses

Statistical analyses were performed using GraphPad Prism 8.0 software. The data are shown as mean ± standard error (SE). Significance of mean differences was determined by two‐tailed Student's *t*‐test. *p < 0.05; **p < 0.01; ***p < 0.001.

## 3 RESULTS

### 3.1 PD‐L1 was increased in highly malignant prostate cancer cell‐derived exosomes

**FIGURE 1:**
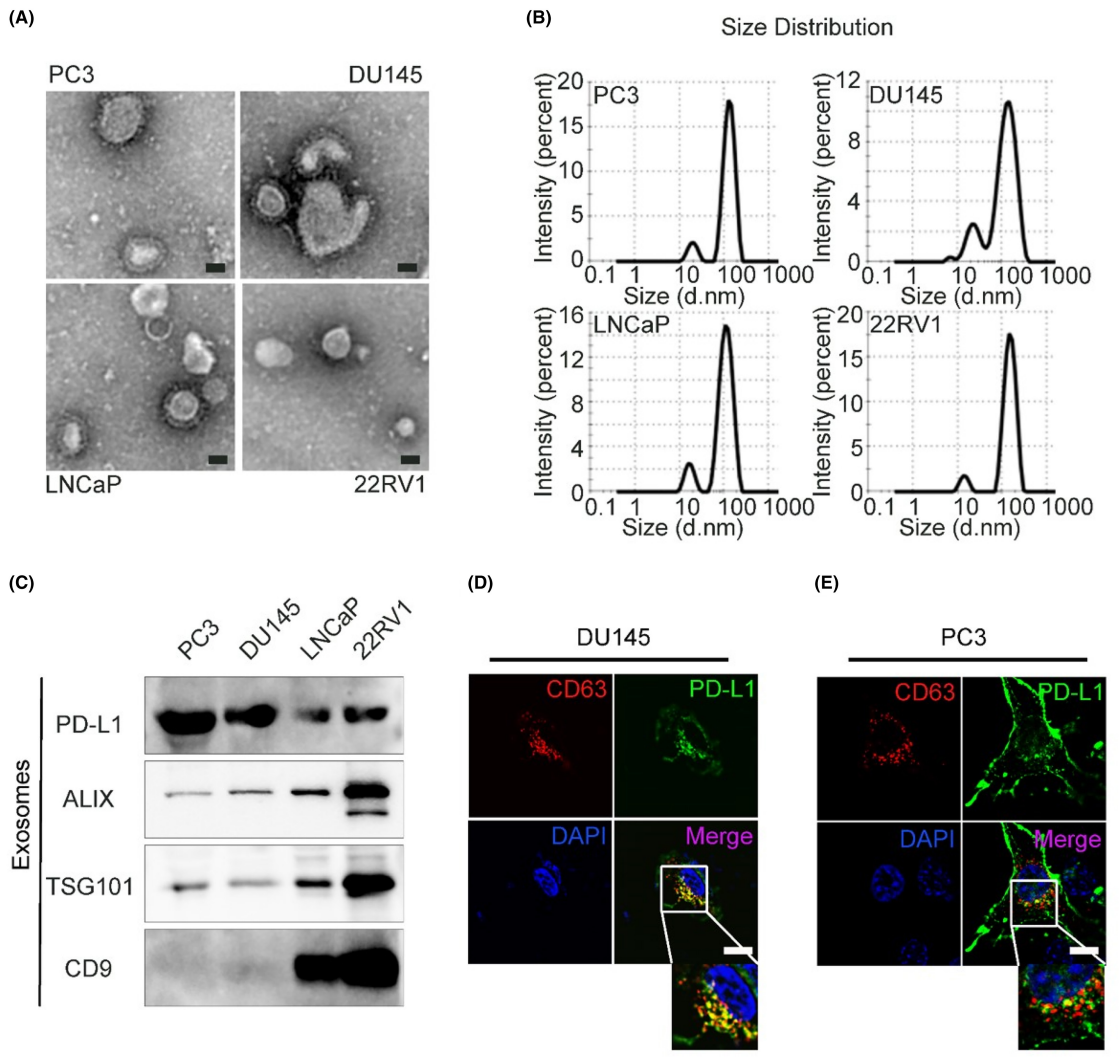
PD‐L1 was increased in exosomes derived from the more malignant prostate cancer cell lines. (A) TEM images of purified exosomes derived from PC3, DU145, LNCaP, and 22RV1 cells; Scale bar represents 50 nm; (B) Size distribution of purified exosomes; (C) Immunoblots for PD‐L1 in the purified exosomes derived from different prostate cancer cell lines. (D, E) Microscopy of PD‐L1‐eGFP (green) and CD63 (red) in DU145 cells (D) or PC3 cells (E); Nuclei (blue); Co‐localization of PD‐L1 and CD63 (yellow); Scale bar represents 10 μm.

To compare the expression levels of PD‐L1 in exosomes from prostate cancer cells with different degrees of malignancy, culture supernatants of various human prostate cancer cell lines (PC3, DU145, LNCaP, and 22RV1) were collected to purify exosomes by ultracentrifugation. Electron micrographs revealed that these exosomes were enveloped by a membrane and exhibited a typical saucer or cup‐like structure (Figure 1A). The particle size distribution showed that the size of the isolated exosomes was approximately 100 nm (Figure 1B). We detected the content of PD‐L1 in exosomes with immunoblot protein assays and found that PD‐L1 was expressed in the exosomes secreted by all four types of PCa cell lines. However, PC3 and DU145 cells expressed more exosomal PD‐L1 than LNCaP and 22RV1 cells (Figure 1C). It is important to note that PC3 and DU145 cells are more aggressive than the latter two types.^25^, ^26^ Immunofluorescence staining revealed that PD‐L1 co‐localized with a well‐established exosome marker (CD63) in PC3 and DU145 cells (Figure 1D,E). Taken together, these data suggest that exosomal PD‐L1 is widely present in prostate cancer cell lines and increases in more malignant cells.

### 3.2 Exosomal PD‐L1 was secreted into the circulatory system

**FIGURE 2:**
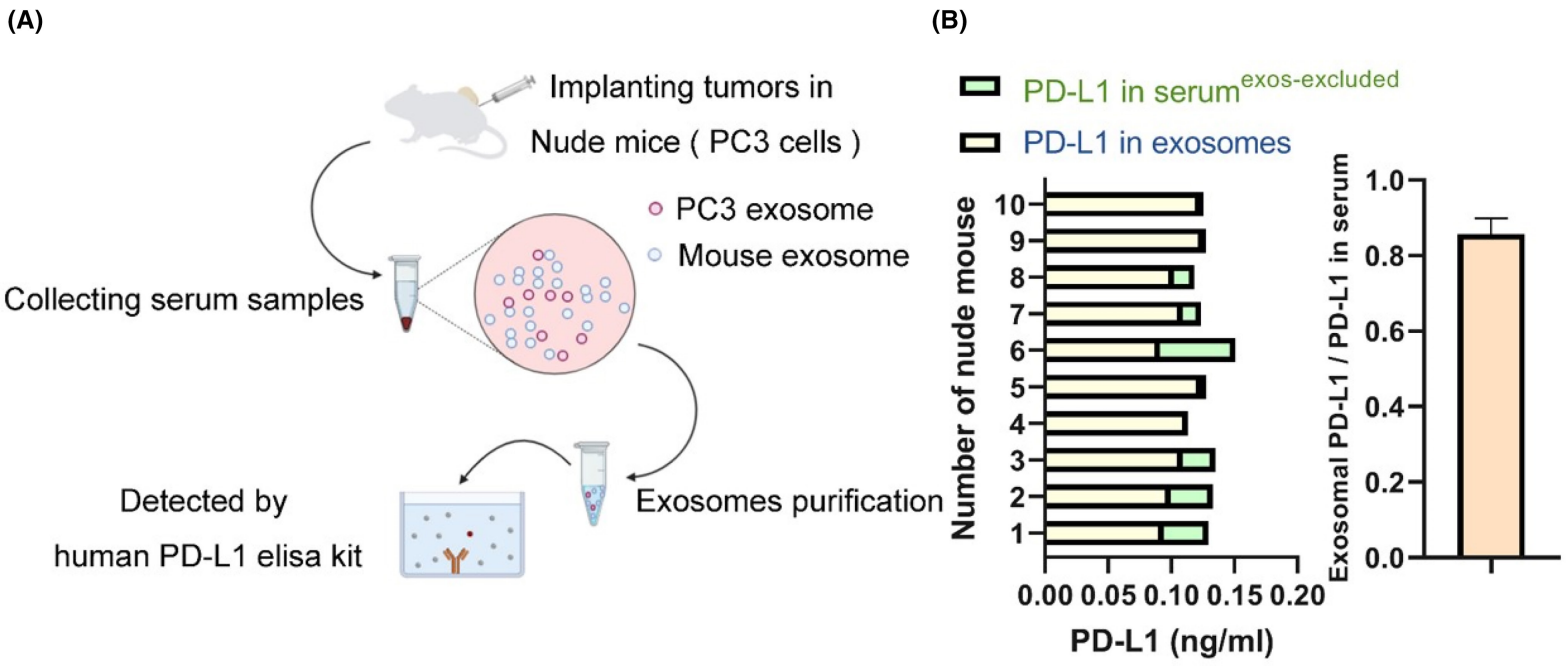
Exosomal PD‐L1 was secreted into the circulation. (A) Schematic of the experimental design. (B) Levels of human PD‐L1 on exosomes purified from serum or in serum as measured by ELISA. Serum samples were obtained from PC3 prostate tumor xenograft‐bearing nude mice.

To evaluate whether prostate cancer cells secrete exosomal PD‐L1 in vivo, we established a subcutaneous xenograft model in nude mice using the PC3 cell line. Blood samples were obtained by cardiac puncture 3 weeks after tumor implantation or when the longest dimension of the tumor reached 2 cm. Exosomes were purified from mouse serum and subsequently used to detect human‐specific PD‐L1 by ELISA assay (Figure 2A). Human‐specific PD‐L1 was detectable in both exosomes and exosome‐depletion sera. However, the content of human PD‐L1 in exosomes accounted for the majority of human PD‐L1 in serum (Figure 2B). These results suggested that prostate tumor cells can secrete PD‐L1 into the circulatory system, mainly in exosome content.

### 3.3 Exosomal PD‐L1 transferred between tumor cells and helped resist T‐cell‐killing

**FIGURE 3:**
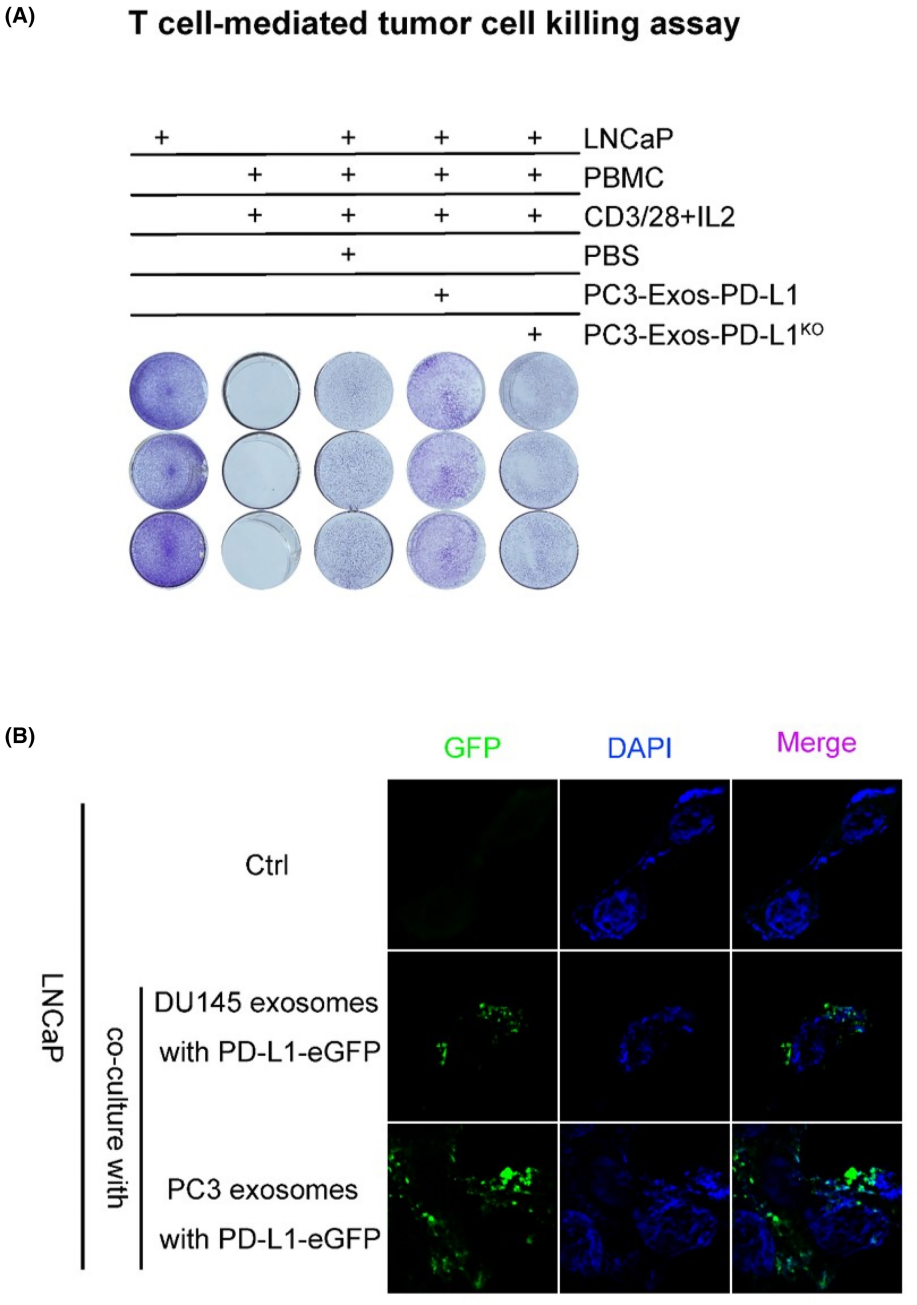
Exosomal PD‐L1 transferred between tumor cells and helped resist T‐cell killing. (A) T‐cell‐mediated tumor cell killing assay; LNCaP cells were co‐cultured with CD8^+^ T‐cells and incubated with PC3 cell‐derived exosome‐PD‐L1, exosome‐PD‐L1KO, or PBS; (B) Microscopy of exosomal PD‐L1‐eGFP (green) in LNCaP cells; Nuclei (blue); LNCaP cells were incubated with exosomes derived from DU145 cells or PC3 cells transfected with pcDNA3.1‐PD‐L1‐eGFP.

The PD‐1 and PD‐L1 interaction may reduce tumor cell death by suppressing T‐cell functions. To evaluate the potential function of exosomal PD‐L1 in the tumor microenvironment, the low‐expressing exosomal PD‐L1 LNCaP cell line was co‐cultured with activated T‐cells and supplemented with exosomes derived from PC3 cells with PD‐L1 knocked out (Exo‐PD‐L1^KO^) or overexpressed (Exo‐PD‐L1^HA^). Results showed that Exo‐PD‐L1^HA^ effectively inhibited the killing effect of T cells on tumor cells, while Exo‐PD‐L1^KO^ did not (Figure 3A). It has been reported that free exosomal PD‐L1 can block T‐cell killing functions directly by binding to PD‐1 on the cell surface of T‐cells. We investigated whether exosomal PD‐L1 can be taken up by tumor cells to protect them from T‐cell killing. To trace PD‐L1, exosomes were purified from the supernatants of PC3 and DU145 cells expressing the PD‐L1‐eGFP fusion protein expression plasmid. After incubation of these exosomes with LNCaP cells, exosomal‐PD‐L1‐eGFP was found to be taken up by the LNCaP cells (Figure 3B, Figure S1). Thus, PD‐L1 can be transferred between tumor cells via exosomes and may help them resist T‐cell killing.

### 3.4 LNCaP cells incubated with exosomes containing PD‐L1 suppress the functionality of CD8 ^+^ T cells

**FIGURE 4:**
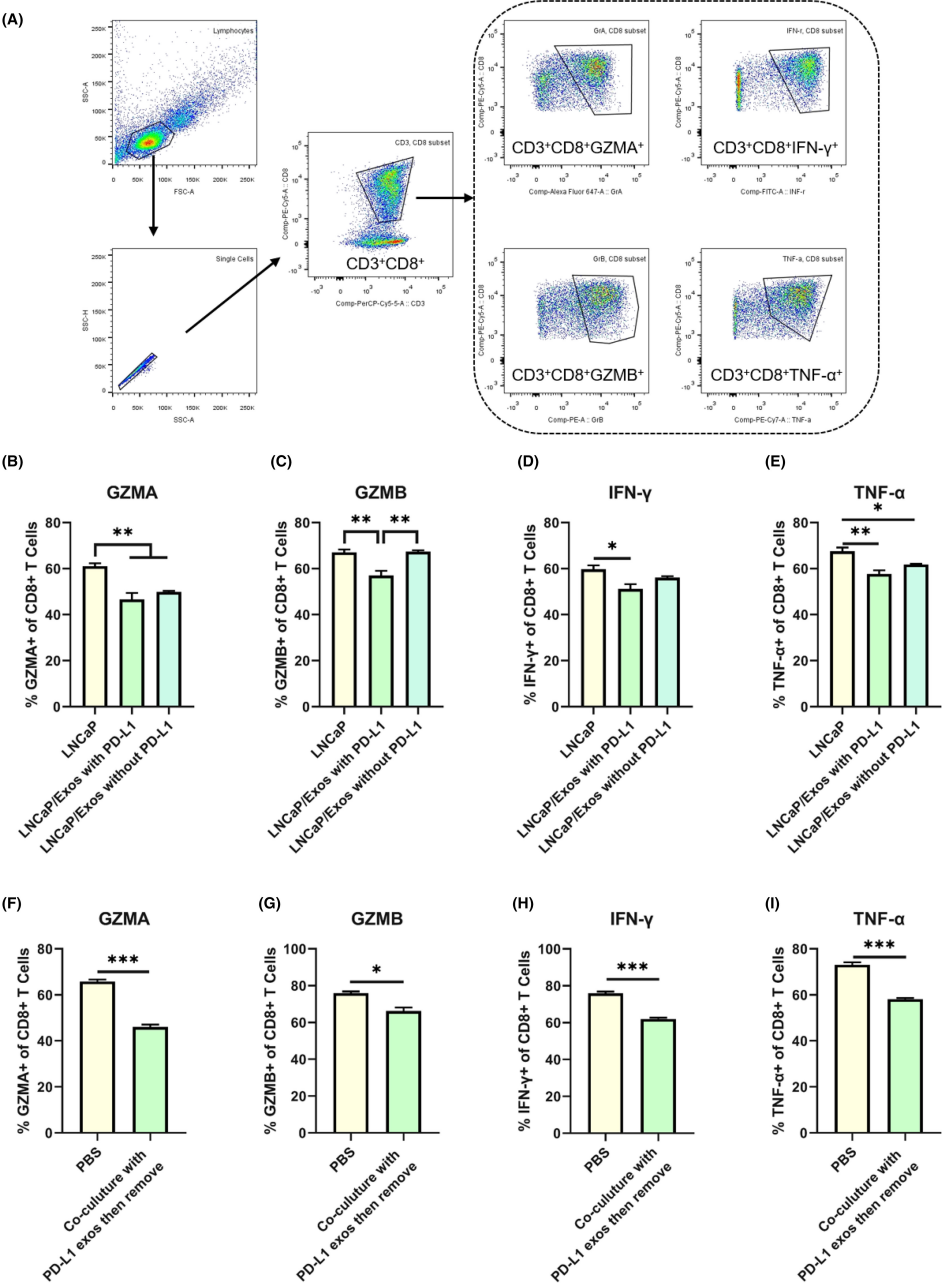
LNCaP cells incubated with exosomes containing PD‐L1 suppress the functionality of CD8^+^ T cells. (A) The analysis strategies for FACS; (B–E) FACS analysis of CD8^+^ T cell functions by detecting cytotoxicity related indicator including Granzyme A (B), Granzyme B (C), IFN‐γ (D), TNF‐α (E). CD8^+^ T cells were co‐cultured with LNCaP cells and incubated with PBS or PC3 cell derived exosomes containing PD‐L1 or not. (F–I) FACS analysis of CD8^+^ T cell functions by detecting cytotoxicity related indicator including Granzyme A (F), Granzyme B (G), IFN‐γ (H), TNF‐α (I). LNCaP cells were incubated with PBS or PC3 cell derived exosomes containing PD‐L1 for 24 h. Then remove the medium containing exosomes and co‐culture with CD8^+^ T cells; *p < 0.05; **p < 0.01; ***p < 0.001.

To assess the effect of exosomal PD‐L1, we detected the functionality of CD8^+^ T cells by flow cytometry analysis (Figure 4A). Compared to co‐culturing with LNCaP only, fewer CD8^+^ T cells expressed cytotoxicity‐related molecules (granzyme A, granzyme B, TNF‐α, and IFN‐γ) in the group of LNCaP incubated with PD‐L1 exosomes (Figure 4B–E, Figure S2). As we have shown above, PCa tumor cells can ingest exogenous exosomes with PD‐L1 and then acquire the ability to resist T‐cell killing. To further prove it, we removed the exosomes containing PD‐L1 after incubating with LNCaP cells for 24 h and then co‐cultured LNCaP cells with CD8^+^ T cells. Flow cytometry analysis indicated that the functionality of CD8^+^ T cells was still suppressed (Figure 4F–I, Figure S3). These results suggest that exosomal PD‐L1 brings the immunity‐evading ability to LNCaPcells, who retain it even after the removal of exosomes from the microenvironment.

### 3.5 Exosomal PD‐L1 promoted the growth of prostate tumors with low PD‐L1 levels in vivo

**FIGURE 5:**
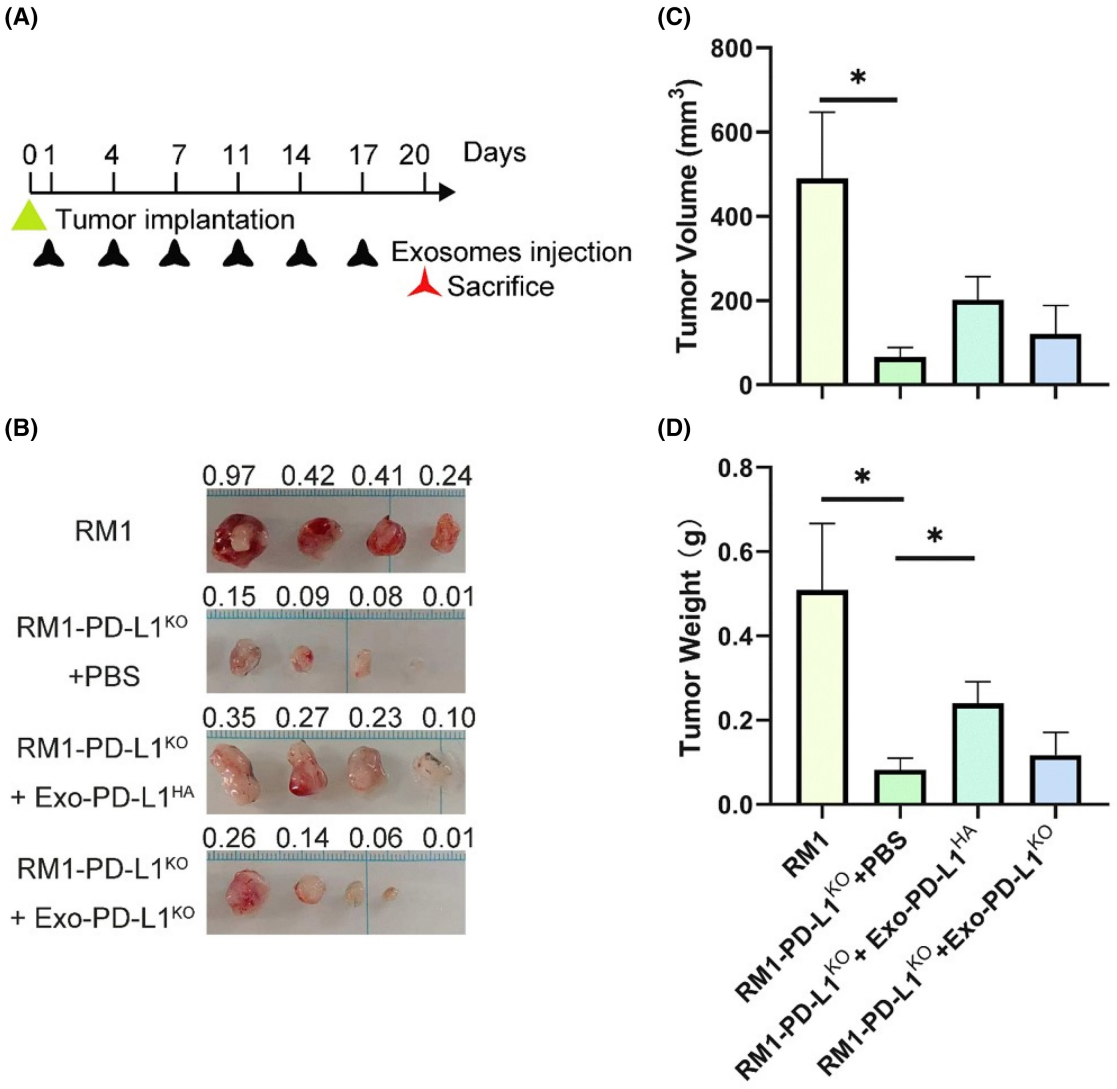
Exosomal PD‐L1 promoted the growth of prostate tumors with low PD‐L1 levels in vivo. (A) Schematic of the experimental design. (B) The tumor images of RM1 cells or PD‐L1 deficient RM1 cells (RM1‐PD‐L1KO) in C57BL/6J mice; Mice implanted with RM1‐PD‐L1KO were injected with exosome‐PD‐L1HA, exosome‐PD‐L1KO, or PBS; Tumor weight is marked above the tumor. (C, D) Tumor volume (C) and tumor weight (D) were measured at 3 weeks after tumor implantation; *p < 0.05.

To evaluate the role of exosomal PD‐L1 in prostate cancer progression, we established a subcutaneous xenograft mouse model using the RM1 mouse prostate cancer cell line with or without PD‐L1 removal. Exosomes with or without PD‐L1 were injected into the tail vein every 3 days (Figure 5A). Depletion of PD‐L1 in RM1 cells inhibited tumor growth compared to the RM1 group. Interestingly, tumor growth was significantly restored after injection of Exo‐PD‐L1^HA^, but not by injection of Exo‐PD‐L1^KO^ (Figure 5B–D), indicating that exosomal‐PD‐L1 promotes tumor growth.

## 4 DISCUSSION

Malignant cells can evade immune elimination through upregulating PD‐L1 on the cell surface, thereby inhibiting T cell function and antitumor immunity.^15^ PD‐L1 expression tends to be increased in advanced and metastatic prostate cancer and is associated with poor prognosis.^27^ The PD‐L1 expression levels were analyzed in a study containing 46 cases of PCa tumor tissues with Gleason score arranged from 5 to 9. PD‐L1 has been found to be positively correlated to Gleason score, indicating a role of PD‐L1 in PCa development. In addition, the alteration of PD‐L1 in tumor tissues was concomitant with elevated RelB, which contributed to the regulation of PD‐L1 expression.^28^ Recently, Poggio et al. found that prostate cancer cell PD‐L1 can be loaded on exosomes to affect distant T cells.^16^ In this study, we also found that highly malignant prostate tumor cells secrete a considerable amount of exosomal PD‐L1 into circulation. Exosomal PD‐L1 helps PCa tumor cells evade the immune system. AR‐negative and more aggressive cell lines PC3 and DU145 secrete more exosomal PD‐L1 than AR‐positive cell lines LNCaP and 22RV1 (Figure 1C). In addition, PD‐L1 expression is closely related to Gleason score and maybe a cofactor associated with PCa progression.^29^ Presumably, high levels of exosomal PD‐L1 may indicate that the PCa tumors are more aggressive. The establishment of this blood‐derived biomarker has the potential to be informative of tumor aggressiveness and be a subject of future research concerning predictive biomarkers for immunotherapy against mCRPC.^30^

Prostate cancer is considered a “cold” tumor, with few immune cells recognized and infiltrating into the tumor microenvironment (TME).^2^, ^5^, ^31^ Accordingly, prostate cancer has been found to respond poorly to immune checkpoint inhibitors.^5^, ^12^, ^13^, ^32^ Turning “cold” into “hot” and enhancing T cell infiltration into the TME are potential strategies to boost antitumor immunity. Prostate tumor cells have been found to suppress T cell activation in draining lymph nodes by secreting exosomal PD‐L1, resulting in systemic immunosuppression. In this study, we also found that prostate tumor cells secrete exosome‐PD‐L1 into the circulation and transfer it between tumor cells. These data suggest that exosomal PD‐L1 may be responsible for the reduced T cell activity in the tumor microenvironment. Therefore, inhibition of exosomal PD‐L1 levels may increase the activity of T cells in the tumor microenvironment and improve patient response to anti‐PD‐L1 therapy.

Accurate screening of responding patients is a great challenge in precision tumor therapy. Although high PD‐L1 is a predictive and prognostic marker, some patients with low PD‐L1 or even negative have been reported to benefit from the therapy. Besides, some tumors develop resistance to immunotherapy despite being sensitive at start.^33^, ^34^ Chen et al. found that exosomal PD‐L1 acted as decoy and bount to anti‐PD‐L1, which was then subsequently cleared more quickly by macrophages, resulting in anti‐PD‐L1 therapy resistance.^17^ In addition, high levels of circulating exosomal PD‐L1 may indicate that the patient's T cells are depleted and anti‐PD‐1 therapy will fail.^35^ These, along with the effects of exosomal PD‐L1 on distant T cell dysfunction,^16^ suggest that patients with high circulating exosomal PD‐L1 levels maybe not be good candidates for anti‐PD‐L1 or anti‐PD‐1 therapy. Thus, exosomal PD‐L1 may be a potential predictive biomarker for anti‐PD‐L1 or anti‐PD1 therapy.

Exosomes are transporters that transfer biologically functional molecules such as DNA, RNA (microRNA, LncRNA, circRNA), proteins, and lipids between cells.^22^, ^23^, ^24^, ^36^ It has been shown that tumor cells regulate fibroblasts, immune cells, and other cells in the TME through exosomes, creating a favorable environment for their seeding, survival, and growth.^20^, ^21^, ^37^, ^38^ In this study, we found that in prostate cancer, cells with low PD‐L1 expression can ingest exosomal PD‐L1 secreted by those cells with high PD‐L1 expression. This confers the ability of cancer cells with low PD‐L1 expression to evade immunity. Prostate cancer cells effectively suppress the function of CD8^+^ T cells through this sharing mechanism. Restriction of exosomal PD‐L1 secretion may enhance the killing effect of CD8^+^ T cells on tumor cells.

Nonetheless, how PD‐L1 is sorted into exosomes remains unknown. It is also unclear why PD‐L1 tends to be loaded onto exosomes in some cancer cells but not in others. However, modifications of PD‐L1, such as palmitoylation, are likely the potential mechanism.^39^, ^40^

In conclusion, we discovered a synergistic defense mechanism among prostate cancer cells. By sharing exosomal PD‐L1, prostate tumor cells acquire immune evasion. Furthermore, exosomal PD‐L1 in sera is a potential marker for prostate cancer diagnosis and prognosis.

## AUTHOR CONTRIBUTIONS

**DaMeng Li:** Conceptualization (equal); data curation (lead); formal analysis (lead); funding acquisition (equal); investigation (lead); methodology (lead); project administration (lead); validation (lead); writing – original draft (lead). **XueYing Zhou:** Data curation (equal); investigation (equal); methodology (equal); validation (equal); writing – original draft (equal). **WenXian Xu:** Data curation (equal); investigation (equal); methodology (equal). **YuXin Chen:** Data curation (equal); investigation (equal); methodology (equal); validation (equal). **ChengLong Mu:** Data curation (equal); methodology (equal). **XinChun Zhao:** Data curation (equal); methodology (equal). **Tao Yang:** Conceptualization (equal); investigation (equal); validation (equal). **Gang Wang:** Data curation (equal); investigation (equal); resources (equal); validation (equal). **Liang Wei:** Conceptualization (lead); funding acquisition (lead); investigation (equal); software (equal); supervision (lead); validation (equal); writing – review and editing (lead). **bo ma:** Conceptualization (lead); data curation (equal); formal analysis (equal); funding acquisition (lead); investigation (lead); methodology (equal); project administration (lead); resources (equal); software (equal); supervision (lead); validation (equal); writing – original draft (lead); writing – review and editing (lead).

## FUNDING INFORMATION

This work was supported by National Natural Science Foundation of China (82073212), the Natural Science Foundation of the Jiangsu Higher Education Institutions of China (22KJA320009), Natural Science Foundation of Jiangsu Province and Jiangsu Planned Projects for Postdoctoral Research Funds (2021K431C).

## CONFLICT OF INTEREST STATEMENT

The authors declare that the research was conducted in the absence of any commercial or financial relationships that could be construed as a potential conflict of interest.

## Supporting information


Figure S1:

## Data Availability

The data that supports the findings of this study are available in the manuscript and its additional files.
