# Supplementary material for: Prostate cancer cells synergistically defend against CD8 + T cells by secreting exosomal PD‐L1
Source: Cancer Med. 2023 Jul 27;12(15):16405–15. doi: 10.1002/cam4.6275 (PMC10469662; doi:10.1002/cam4.6275)
Supplement: Supplementary file 1 — Figure S1: [file CAM4-12-16405-s001.docx]

Figure S1


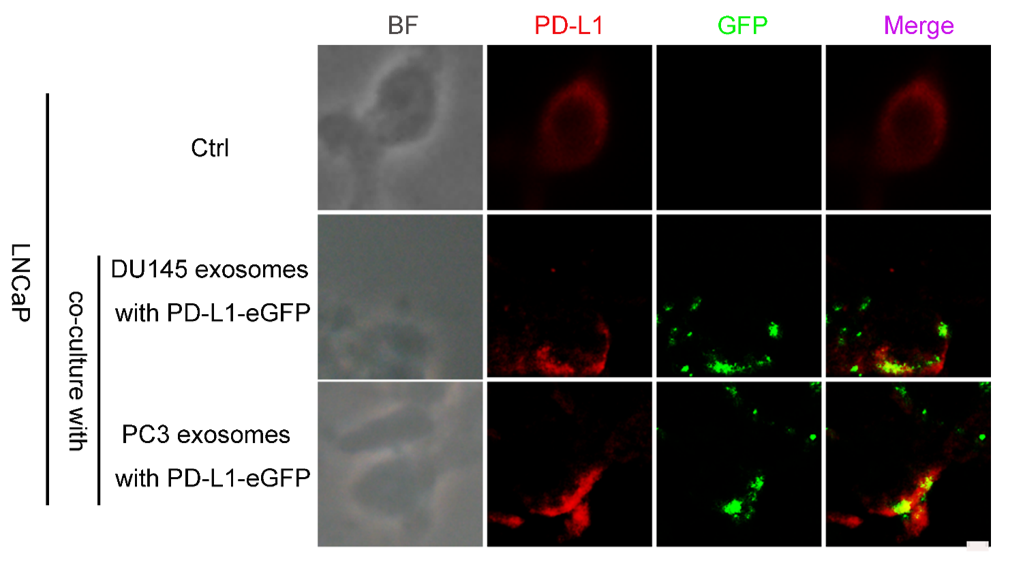


Figure S1: PD-L1 staining in LNCaP cells after exosomes from DU145 or PC3 cells have been added (related to Figure 3B). LNCaP cells were treated with exosomes from DU145 cells and PC3 cells transfected with pcDNA3.1-PD-L1-eGFP. Microscopy of PD-L1 (Red; stained with PD-L1 antibody) and PD-L1-eGFP (Green) in LNCaP cells; BF: bright field.

Figure S2


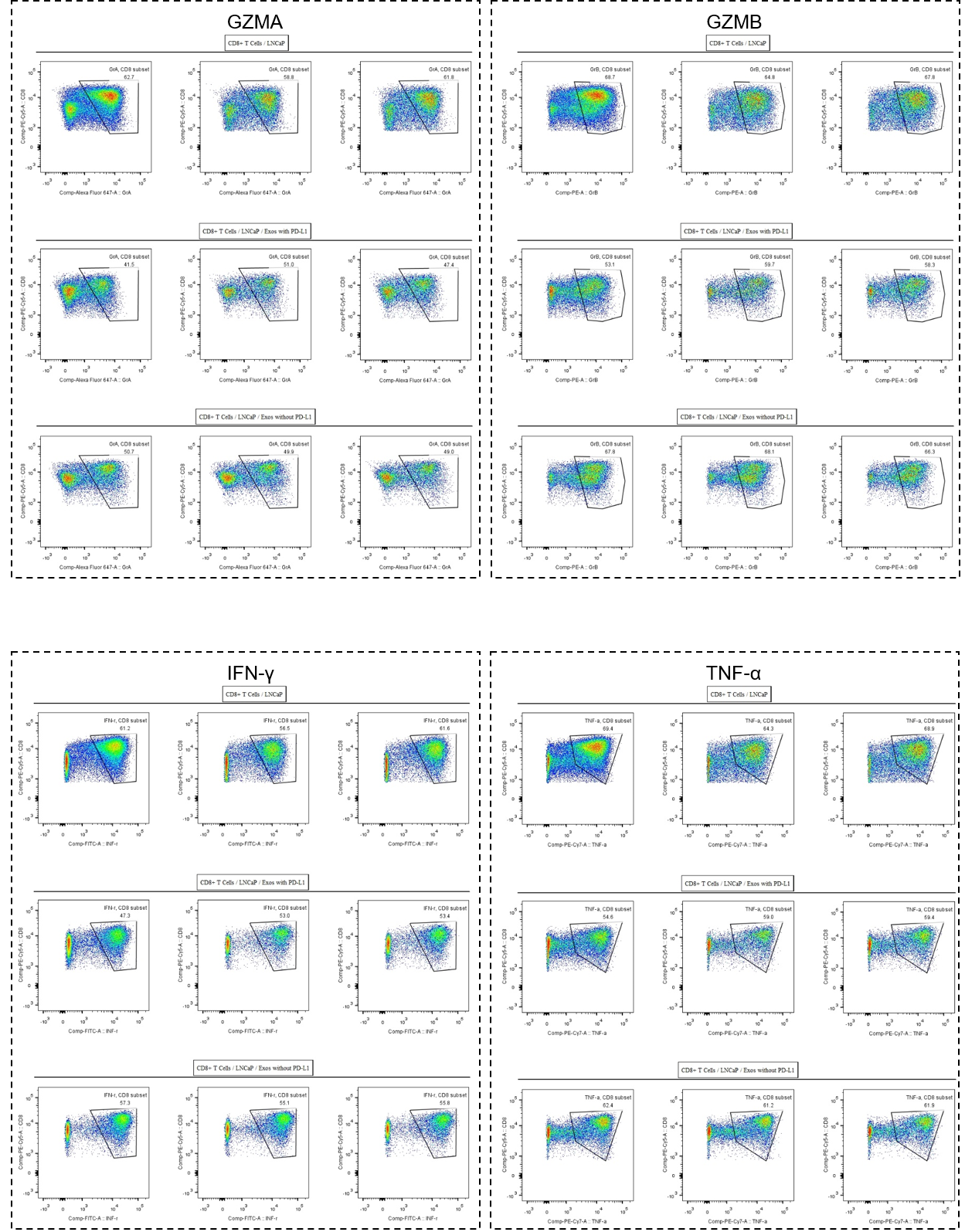


Figure S2: Functional analysis of CD8+ T cell by flow cytometry (related to Figure 4B-4E). Granzyme A, Granzyme B, IFN-γ and TNF-α were detected. Top panel: CD8+ T cells were co-cultured with LNCaP cells and PBS; middle panel: CD8+ T cells were co-cultured with LNCaP cells and incubated with PC3 cells derived exosomes containing PD-L1; bottom panel: CD8+ T cells were co-cultured with LNCaP cells and incubated with exosomes derived from PD-L1^KO^_PC3 cells.

Figure S3


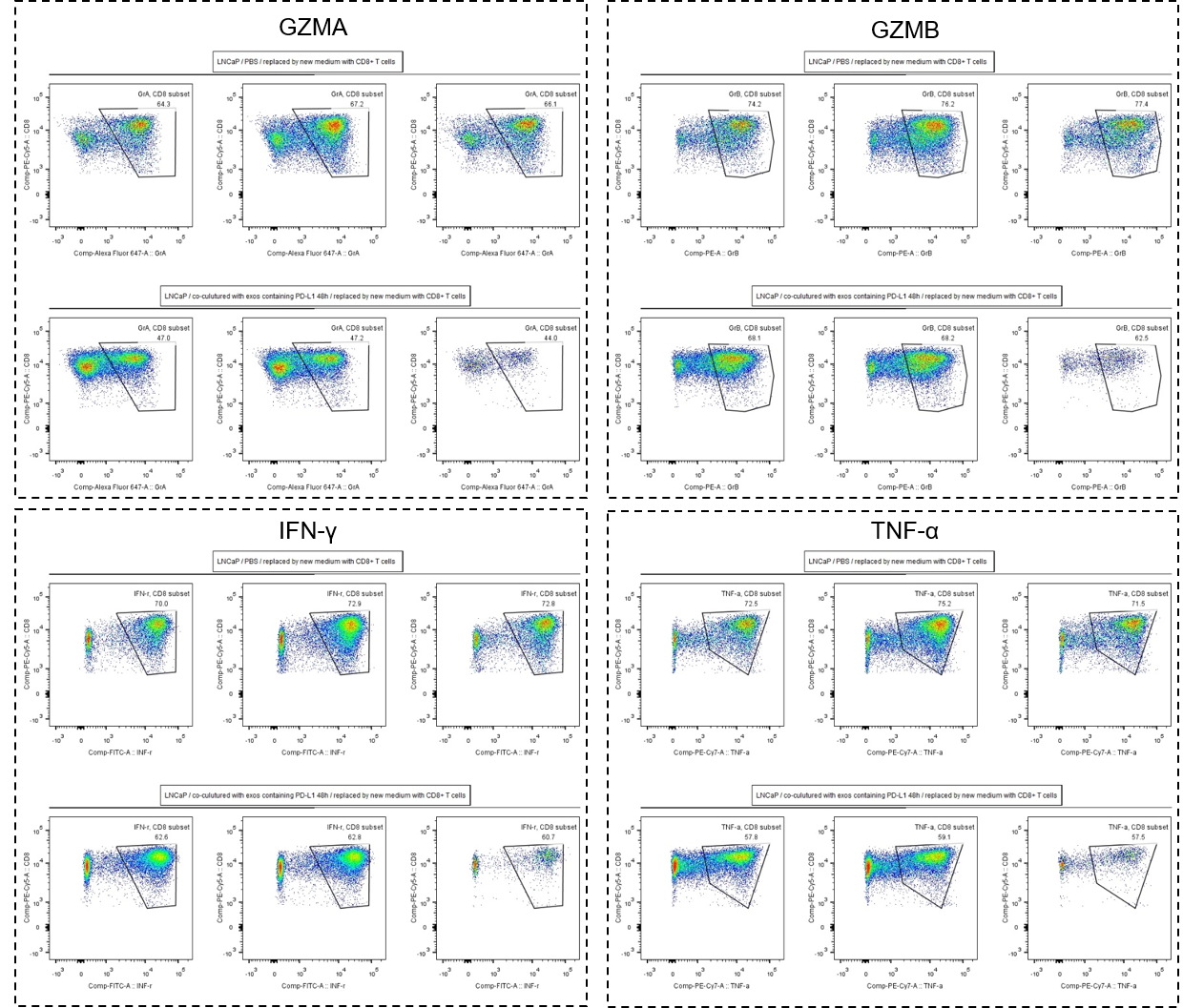


Figure S3: Functional analysis of CD8+ T cell (related to Figure 4F-4I). FACS analysis of CD8+ T cell functions by detecting Granzyme A, Granzyme B, IFN-γ and TNF-α. Top panel: CD8+ T cells were co-cultured with LNCaP cells and PBS; bottom panel: LNCaP cells were incubated with PC3 cells derived exosomes containing PD-L1 for 24h, then removed the medium containing exosomes and co-cultured with CD8+ T cells.
